# Association of NOD2 and IL23R with Inflammatory Bowel Disease in Puerto Rico

**DOI:** 10.1371/journal.pone.0108204

**Published:** 2014-09-26

**Authors:** Veroushka Ballester, Xiuqing Guo, Roberto Vendrell, Talin Haritunians, Alexandra M. Klomhaus, Dalin Li, Dermot P. B. McGovern, Jerome I. Rotter, Esther A. Torres, Kent D. Taylor

**Affiliations:** 1 Department of Medicine, Division of Gastroenterology, School of Medicine, University of Puerto Rico Medical Sciences Campus, San Juan, Puerto Rico; 2 Medical Genetics Institute & Inflammatory Bowel Disease Center, Cedars-Sinai Medical Center, Los Angeles, California, United States of America; 3 Institute of Translational Genomics and Population Sciences, Los Angeles Biomedical Research Institute at Harbor/UCLA Medical Center, Torrance, California, United States of America; CWRU/UH Digestive Health Institute, United States of America

## Abstract

The Puerto Rico population may be modeled as an admixed population with contributions from three continents: Sub-Saharan Africa, Ancient America, and Europe. Extending the study of the genetics of inflammatory bowel disease (IBD) to an admixed population such as Puerto Rico has the potential to shed light on IBD genes identified in studies of European populations, find new genes contributing to IBD susceptibility, and provide basic information on IBD for the care of US patients of Puerto Rican and Latino descent. In order to study the association between immune-related genes and Crohn’s disease (CD) and ulcerative colitis (UC) in Puerto Rico, we genotyped 1159 Puerto Rican cases, controls, and family members with the ImmunoChip. We also genotyped 832 subjects from the Human Genome Diversity Panel to provide data for estimation of global and local continental ancestry. Association of SNPs was tested by logistic regression corrected for global continental descent and family structure. We observed the association between Crohn’s disease and NOD2 (rs17313265, 0.28 in CD, 0.19 in controls, OR 1.5, p = 9×10^−6^) and IL23R (rs11209026, 0.026 in CD, 0.0.071 in controls, OR 0.4, p = 3.8×10^−4^). The haplotype structure of both regions resembled that reported for European populations and “local” continental ancestry of the IL23R gene was almost entirely of European descent. We also observed suggestive evidence for the association of the BAZ1A promoter SNP with CD (rs1200332, 0.45 in CD, 0.35 in controls, OR 1.5, p = 2×10^−6^). Our estimate of continental ancestry surrounding this SNP suggested an origin in Ancient America for this putative susceptibility region. Our observations underscored the great difference between global continental ancestry and local continental ancestry at the level of the individual gene, particularly for immune-related loci.

## Introduction

Meta-analyses of genome-wide association studies (GWAS) by an international effort have now identified over 160 genomic regions contributing to the inflammatory bowel diseases (IBD), Crohn’s disease (CD) and ulcerative colitis (UC) in populations of European descent.[1]–[3] Many of these genes overlap with genes for other immune-related traits such as psoriasis and ankylosing spondylitis.

Studies of populations of African and Asian descent suggest that the study of IBD genetics in non-European populations may show: 1) same gene, same SNPs, but stronger effects than what has been observed in populations of European descent, for example TNFSF15; [4], [5] 2) same gene, different SNPs than what has been observed in populations of European descent, for example NOD2; [6] as well as 3) different gene, different SNPs contributing to susceptibility. [7] When taken together with the large number of mouse models of intestinal inflammation, these human results support the concept that multiple pathways lead to intestinal inflammation, and that, just as in different mouse strains, different combinations of susceptibility loci may act in different human populations. [8], [9].

Populations from three continents have contributed to the current genetic composition of Puerto Rico. Prior to the European explorations of the 16^th^ century, there were at least four waves of Native American migration to the island, interspersed with extensive periods of intermixture across the Caribbean. The last of these, the Taíno population, was present on the island at the time of Spanish colonization. [10] Concomitant with European colonization, the Taíno suffered a genetic bottleneck from extensive deaths by disease, particularly influenza and smallpox, by the harsh conditions of slavery, and by warfare with the European colonists. [11] Import of slaves from West Africa began in 1513 in order to keep the sugar industry economically viable; in the same year Spanish citizens were granted the right to marry Taíno by the Spanish crown. Multiple migrations of both Puerto Ricans and of Africans have occurred back and forth across the Caribbean up to the present day. Thus, the population of Puerto Rico may be modeled as an admixed population with contributions from three continental areas: Sub-Saharan Africa, from the slave trade, America, from the extensive migrations of Native Americans prior to colonization, and Europe, from the colonization of the “New World” by European powers. [12], [13].

This admixture affords a unique opportunity for IBD gene identification. Three continental ancestries undergoing different selection pressures have the potential to create genomic combinations that may augment or diminish the effect of a given locus. Since the Puerto Ricans have European ancestry, different combinations may shed light on loci already identified in the extensive genetic studies of IBD in populations of European descent. While discovered in European populations, the effect of some of these may be easier to study in an admixed population. In addition, new IBD loci may also be detected; identification of these is important not only for Puerto Rico, but for all US urban centers with admixed populations.

The ImmunoChip was developed by a consortium of investigators studying the genetics of immune-related disorders, including the International IBD Genetics community. Genotyping with this chip using Illumina technology provided the opportunity to obtain an initial examination of already identified IBD genes in the Puerto Rico population as well as to study genes associated with other immune-related disorders at a cost significantly less than that for a genome-wide association study (Illumina, San Diego, CA). [3], [14].

## Methods

### Subjects from Puerto Rico

The subjects in this study were recruited between 2002 and 2011 and are summarized in Table 1. Puerto Ricans with an established diagnosis of Inflammatory Bowel Disease (IBD; Crohn’s Disease, CD, 406; Ulcerative Colitis, UC, 244; Indeterminate Colitis, 5) were recruited from the University of Puerto Rico Center for Inflammatory Bowel Diseases as well as IBD support groups and community gastroenterology practices as part of the NIDDK IBD Genetics Research Consortium.[15]–[18] Recruitment was not focused on any specific clinical features of IBD. Control samples were also collected from a) both parents of an IBD patient, without regard to their IBD status (81 CD families, 39 UC families) or b) spouse or friends of the same age and geographic area. In order to increase the homogeneity of our genetic sample, all patients were required to have both parents and all grandparents with Puerto Rican descent. Data on disease characteristics were extracted from surgical, endoscopic, radiological, and histopathological reports on medical records. Blood samples of 51 ml were obtained from each subject and sent to the Cedars-Sinai Medical Center Genetics for isolation of serum, extraction of DNA, and establishing of EBV-transformed lymphoblastoid cell lines. This study was approved by the Institutional Review Boards of the University of Puerto Rico, the Los Angeles Biomedical Research Institute, and the Cedars-Sinai Medical Center.

**Table 1:** Description of study subjects.

Category	Crohn’s Disease	Ulcerative Colitis	Indeterminate Colitis	Not Affected
CASE CONTROL ANALYSES	403	240	5	274
GWAF ANALYSES	406	244	5	504
DETAILS OF SUBJECTS				
Number of families	80	39		
Number index cases in families	80	39		
Number of additional affecteds in families	3	4		
Number of unaffected in families				230
Number of affecteds not in families	323	201	5	
Number of unaffecteds not in families				274
Proportion of females	0.48	0.55		
Mean age at diagnosis (yr)	26	31		

### Subjects from the Human Genome Diversity Project

DNA samples for the subjects in the Human Genome Diversity Project (HGDP) were obtained from CEPH. This project has been described elsewhere, including the consenting methods and approvals for the various populations in this panel.[19]–[21] Additional DNA samples were obtained from the Coriell repository: Karitiana-Rondonia and Surui-Rondonia of Brazil (5 samples each); Mayan-Campeche of Yucatan (2 samples); Pima of Northwest Mexico (5 samples); Quecha of Andes (5 samples); other Andes (5 samples); Auca of Ecuador (1 sample); and an African panel (Yoruban, Biaka, Mbuti, Bantu). Overlap with the CEPH-HGDP was identified upon genotyping and removed. Use of these DNAs as de-identified population controls in this project was also approved by the Institutional Review Boards of the University of Puerto Rico, the Los Angeles Biomedical Research Institute, and the Cedars-Sinai Medical Center.

### Genotyping

DNA was isolated from approximately one million cells from the EBV-transformed cell lines using Qiagen columns following the manufacturer’s instructions (Qiagen, Valencia, CA). Genotyping was performed using the ImmunoChip (Illumina, San Diego, CA) developed by an international consortium of investigators studying major immune-related diseases such as Crohn’s disease, ulcerative colitis, rheumatoid arthritis, ankylosing spondylitis, systemic lupus erythematosus, autoimmune thyroid disease, celiac disease, and multiple sclerosis. [14] Because the iChip is a custom designed chip and the Puerto Rican and HGDP subjects are very genetically diverse, quality control included testing for overall intensity of dye reactions, for balance between both dyes in this two-dye system, for differential missing data across genotyping runs, for adequate separation between genotype clusters as well as number of clusters, for Hardy-Weinberg equilibrium, and for abnormal cluster patterns indicating missing alleles or other assay problems due to genetic variation across the various populations. Monomorphic SNPs or SNPs with a minor allele frequency (MAF) less than 0.005 were also removed. Over 12,000 cluster plots were manually reviewed for this study, many by more than one investigator. As a final quality control measure, all SNPs in this report have been reviewed again prior to publication. A dataset of 1159 Puerto Rican and 832 HGDP samples for 155,868 SNPs was available to this study.

### Estimate of “global” or “genomic” continental ancestry

The SNP dataset was thinned to 20,812 SNPs without pairwise linkage disequilibrium using PLINK. [22] Principal components analysis was conducted following standard methods. [23], [24] The proportion of ancestry from populations from Africa, Europe, and Ancient America (Mexico, Central, and South America) was determined for each Puerto Rican subject by performing a supervised analysis using Admixture 1.22. [25], [26] We defined “Africa” by subjects from the Bantu, Biaka, Mandenkan, Mbuti, San, and Yoruban populations (AFR; 126 subjects), “Europe” by subjects from the Adygei, Basque, French, Italian, Orcadia, Russia, Sardinia, and Tuscan populations (EUR; 136 subjects), and “America” by subjects from the Andean, Auca, Karitiana, Mayan, Piapoco, Pima, Quecha, and Surui populations (AMR; 98 subjects). Note that in this paper, “America” is an abbreviation to refer to the terms “Ancient Americans,” “Native Americans” or “Meso-Americans.” The estimates for African and American continental proportions were then used as covariates to correct for global admixture in the analyses described below.

### Association analyses

In order to use as many of the cases, controls, and family members in this study as possible, association of each SNP with disease was tested by fitting an additive logistic regression model via Generalized Estimating Equations (GEE), with correction for familial correlation and for African and American continental ancestry. This method has been implemented in R as the GWAF package. [27] For convenience, Haploview v4.2 [28] was also used to examine haplotype relationships and linkage disequilibrium, though this program does not allow for the necessary covariate adjustments for this study. We therefore report the p-values from these studies only for making relative comparisons between haplotypes. When frequencies of alleles or haplotypes are reported, the family members were excluded from the determination.

### Estimate of “locus” or “local” continental ancestry

The continental origin of a locus was estimated using a multi-step, locus-specific ancestry method specifically designed for three-way admixed populations (LAMP-LD). [29], [30] For this analysis, African (AFR), American (AMR) and European (EUR) continental groups were defined as above. (1) SNP data for each locus was extracted from the HGDP+Coriell samples separately for the European, American, and African continental populations. (2) Haplotypes for the given locus were reconstructed using fastPHASE,[31]–[33] again keeping the three continental groups separate. (3) SNP data for the locus was extracted separately for Puerto Rican cases and controls and processed using Perl scripts for input into LAMPLD. (4) LAMPLD was applied to the case and control data along with the three continental haplotype sets for training the algorithm. Window size was set at 50 and number of states at 15, reflecting the recommendations of the authors in their paper for the Puerto Rican population. [30] On the whole, separating cases and controls did not result in major differences in local ancestry, so these were combined for plotting in the figures.

## Results

The Puerto Rican subjects genotyped in this study are summarized in Table 1. 1159 Puerto Rican subjects and 832 subjects from the Human Genome Diversity Project (HGDP) were genotyped for 155,868 SNPs with a call rate for each SNP greater than 98% across all samples. Genotype concordance between duplicate samples was greater than 99.999%. Extensive manual review of cluster plots was necessary due to the multiple populations represented in the subjects.

### “Global” continental ancestry

**Figure 1:**
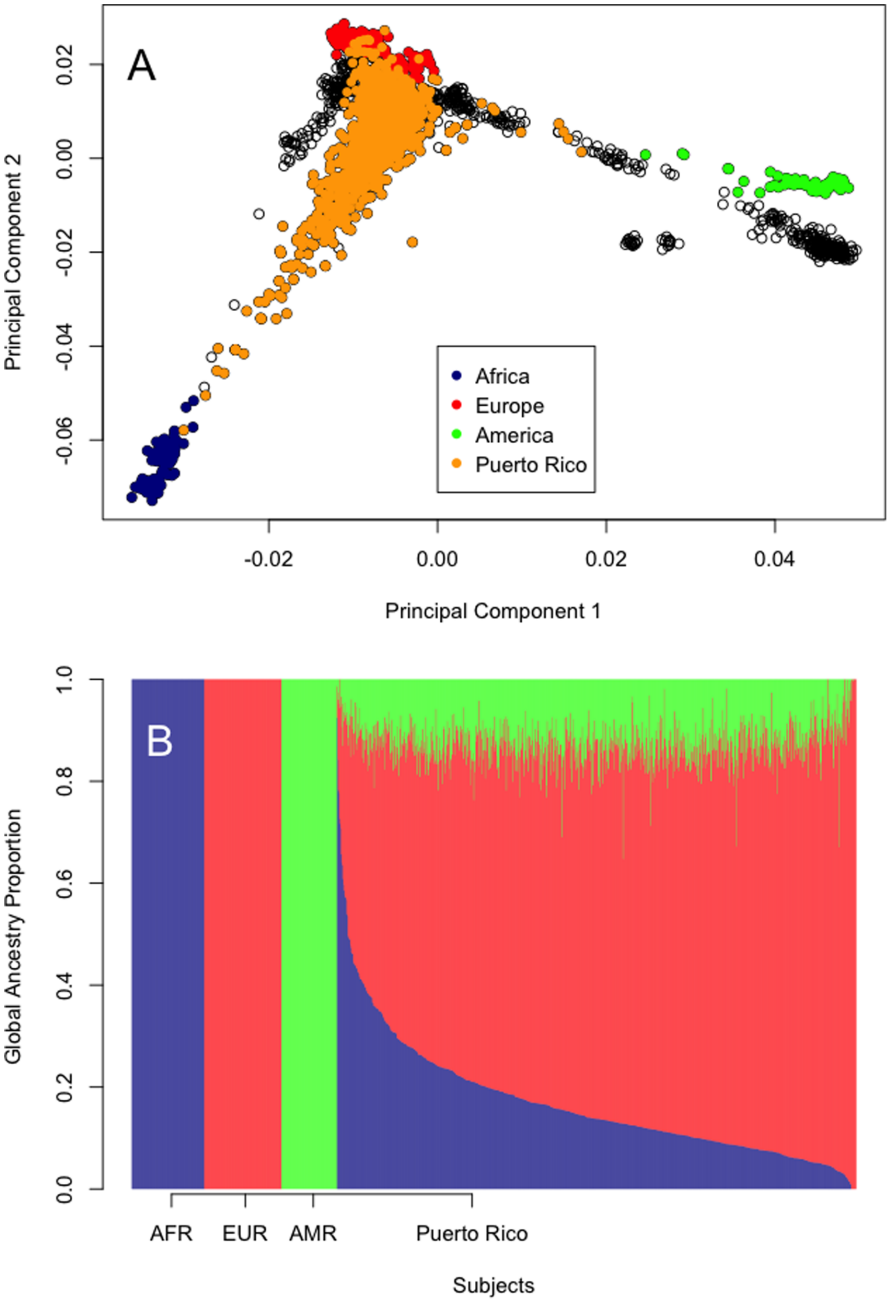
“Global” continental ancestry in Puerto Rican subjects. A. Principal components analysis of the combined Puerto Rican and HGDP subjects. B. “Global” continental ancestry was estimated using Admixture 1.2 in an analysis supervised by data from HGDP populations as described in Methods (AFR, sub-Saharan African continent, dark blue; EUR, European continent, red; and AMR, Mexico, Central and South America continents, green). HGDP subjects not originating from these three continents are black. Puerto Rican subjects were sorted left-to-right based on ancestry from the African continent.

The SNP data were pruned for pairwise linkage disequilibrium and principal components analysis was conducted using 20,812 SNPs. Data from the entire HGDP was included in this analysis. As expected from previous work and from the history of Puerto Rico, the subjects in this study had a mixture of African (AFR, dark blue), European (EUR, red), and American (AMR or green) ancestry (Figure 1a). HGDP subjects not originating from these three continents are shown in black. Global continental ancestry was estimated for each subject using Admixture 1.2 in a supervised analysis. Haplotypes with African, European, and American continental ancestry were modeled using data from Sub-Saharan African, European, and American populations pooled as defined in Methods (Figure 1b). In addition to the modeled ancestries of the controls, Figure 1b also shows the proportion of these three continental ancestries for all of the Puerto Rican subjects, sorted from left to right by proportion of ancestry from West Africa. The median ancestries across all Puerto Rican subjects were 14% African, 74% European, and 12% American. The proportions of African and American ancestry for each subject were then used as covariates in the association analyses to correct for population stratification.

### Association with previously identified IBD loci

Because the Puerto Rican subjects included family members, affected cases, and unrelated controls, we performed a logistic regression analysis for each SNP, correcting for family structure and African and American global continental ancestry using GWAF. This method makes use of most of the available data. We first examined the SNPs that have been previously identified for CD, UC, or IBD in European GWAS studies (Table 2). [3].

**Table 2:** Association of known IBD SNPs.

SNP	Minor allele	Region	Trait	Genes	Minor allele frequency		Association		
					Affected	Unaffected	Beta	Odds ratio	P value
rs734999	C	1p36.32	UC	TNFRSF14	0.52	0.47	0.31	1.36	0.0023
rs3806308	A	1p36.13	UC	OTUD3	0.55	0.46	0.35	1.42	0.0011
rs11209026	A	1p31.3	CD	IL23R	0.026	0.071	−0.89	0.41	0.00038
rs9501161	A	6p21.3	CD	MHC	0.13	0.20	−0.49	0.61	0.000044
rs17582416	G	10p11.21	CD	CUL2	0.36	0.31	0.29	1.34	0.0020
rs8005161	T	14q31.3	UC	GPR65	0.23	0.17	0.50	1.65	0.00026
rs17313265	T	16q12.1	CD	NOD2	0.28	0.19	0.43	1.54	9.3×10–6
rs2836878	A	21q22.2	UC	PSMG1	0.22	0.26	−0.40	0.67	0.0021

“Previously reported SNPs” are those IBD snps added to the design of the ImmunoChip; one per locus is shown here. Since 2 phenotypes were tested for this table (CD and UC) and 100 of the SNPs were not in linkage disequilibrium in Puerto Rico controls, the Bonferroni correction for multiple comparisons is 0.05/(2*100) = 0.00025.

#### NOD2

Puerto Rican CD was associated with NOD2 SNP rs17313265 (Table 2). The haplotype structure of NOD2 (dbGene 64127) in Puerto Rico was similar to that in European populations in that three disease predisposing variants, SNP8 (rs2066844), SNP12 (rs2066845), and SNP13 (rs5743293), are each in linkage disequilibrium with either SNP5 (rs2066842) or SNP6 (rs2066843; Table 3). [34] The association was mainly in NOD2 (Figure S1A, upper, in File S1); a conditional analysis demonstrated that all of the association observed at this locus in Puerto Ricans was explained by the European SNPs (data not shown). None of the three European IBD susceptibility haplotypes were present in HGDP Africans, and only one was observed in HGDP Americans (Table 3). The local continental ancestry at NOD2 in Puerto Ricans was mostly European with very little African ancestry (Figure S1A, lower, in File S1).

**Table 3:** NOD2 haplotypes.

NOD2 Haplotype					Puerto Rico			Human Genome Diversity Panel		
SNP5	SNP6	SNP8	SNP12	SNP13	CD	No IBD	pvalue	Europe	Africa	America
C	C	C	G	D	0.73	0.81	0.006	0.69	0.92	0.93
C	T	C	G	D					0.053	
T	T	C	G	D	0.13	0.13		0.23	0.023	0.056
T	T	**T**	G	D	0.075	0.040	0.056^1^	0.033		
T	T	C	G	**I**	0.038	0.012	0.029^1^	0.029		0.015
T	T	C	**C**	D	0.020	0.004	ns^1^	0.015		

Haplotypes were determined using Phase v2.3; SNPs are, in order, rs2066842 (SNP5), rs2066843 (SNP6), rs2066844 (SNP8), rs2066845 (SNP12), and rs5743293 (SNP13). The association of each haplotype was tested by permutation.

Footnotes:

1) IBD susceptibility haplotype in Europeans.

#### IL23R

CD in Puerto Rico was associated with the previously reported non-synonymous IL23R R381Q SNP (dbGene 149233; rs11209026, Table 2). A conditional analysis showed that this SNP accounts for the association in this genomic region in Puerto Ricans (data not shown). The association was confined to the IL23R gene (Figure S1B, upper, in File S1) and the local continental ancestry was entirely European (Figure S1B, lower, in File S1).

#### Major Histocompatibility Complex (MHC)

CD was associated with rs9501161 located in the NELFE gene (also known as RDBP, dbGene 7936). The complicated linkage disequilibrium structure of the MHC is well-known such that the association is not localized by this observation.

#### GALC/GPR65

An association with UC was also observed in the GALC/GPR65 region (Table 2; Figure S1C, upper, in File S1). The association was observed in a region with increased American and decreased European ancestry (Figure S1C, lower, in File S1).

### Association with other loci included on the ImmunoChip

There were additional associations with SNPs on the ImmunoChip contributed by consortia representing diseases other than IBD.

#### LCE complex

Multiple associations with IBD combined were observed in part of a complex of “late cornified envelope” genes with a peak at LCE1E (dbGene 353135; Table 4; Figure S2A, upper, in File S1). This association was located in a region of predominantly African continental ancestry (Figure S2A, lower, in File S1).

**Table 4:** Top Associations with Additional SNPs.

					Minor Allele Frequency		Minor Allele Association		
Phenotype	SNP	Minor Allele	Locus	Gene(s)	Affected	Not Affected	Beta	Odd Ratio	p value
IBD	rs950337	C	1q21.3	LCE1E; LCE1B	0.24	0.35	−0.36	0.70	2.7×10^−5^
UC	rs1752166	A	9q33.3	DENND1A	0.28	0.24	0.61	1.84	2.0×10^−6^
CD	rs1200332	T	14q13.2	BAZ1A	0.45	0.35	0.43	1.54	2.3×10^−6^
UC	rs9935954	T	16q23.1	CFDP1	0.21	0.12	0.61	1.84	2.1×10^−5^

Associations with pvalues <5×10^−5^ are listed here. Of the ∼115,000 SNPs tested for this table, ∼20,000 SNPs are not in linkage disequilibrium. Since 3 phenotypes were tested, the Bonferroni correction for multiple comparisons is 0.05/(20,000×3) = 8×10^−7^.

#### DENND1A

A peak association with UC was observed at rs677987 in DENND1A (dbGene 57706; Table 4) with additional associations at rs2041545 and rs677987 (beta 0.56, p = 1.2×10^−5^). However, there were not enough SNPs on the ImmunoChip to perform the continental ancestry analysis for this gene.

#### BAZ1A

An association with CD was observed for the promoter of BAZ1A at a level of significance of 2.3×10^−6^ in the GWAF (dbGene 11177; Table 4; Figure S2B, upper, in File S1). When corrected for multiple comparisons, this level is not genome-wide significant (10^−8^), nor significant after correction for the number of SNPs studied (0.05/155,868 = 3.2×10^−7^), nor after correction for the number of independent signals in the SNP data (0.05/approximately 25,000 when pruned for linkage disequilibrium times 3 phenotypes = 6.7×10^−7^). However, the GWAF qq-plot suggested some deviation from the null for rs1200332 (red point, Figure S3, in File S1). The continental origin of this genomic region was predominantly American (Figure S2B, lower, in File S1).

#### BCAR1/CFDP1/TMEM170A

Multiple associations with UC were observed across the region spanning BCAR1 to TMEM170A (gene (BCAR1, dbGene 9564; CFDP1, dbGene 10428; TMEM170A, dbGene 124491; Table 4; Figure S2C, upper, in File S1). The continental origin of this genomic region was predominantly European (Figure S2C, lower, in File S1).

## Discussion

In this study we have genotyped a cohort of subjects from Puerto Rico comprising Crohn’s disease (CD), ulcerative colitis (UC), and “No IBD” controls with the Illumina ImmunoChip (iChip, Illumina, San Diego, CA). In order to provide data for estimating the continental ancestry of interesting regions, we genotyped 832 subjects of the Human Genome Diversity Panel (HGDP) with the same chip at the same time. The design of the iChip has allowed the testing of the association between inflammatory bowel disease (IBD) and genes previously associated with IBD in European populations. [3] In addition, we have been able to test other immune-related genes contributed by consortia studying other immune-related diseases. [14] Since ascertainment of subjects has followed both a case/control design and a family design, we have employed logistic regression corrected for family structure in order to make use of most of the available data (GWAF). Furthermore, we have trained our estimates of continental ancestry using the HGDP data. These estimates are a first step to understanding the additional genetic association in a three-way admixed population and our observations underscore the concept that “local” continental ancestry at a given locus may be very different from “global” continental ancestry across a population, particularly for genes related to the human immune system.

We observed the association of genes originally identified in European populations in Puerto Rican CD: NOD2, IL23R, the MHC, and GALC/GPR65. The NOD2 haplotype structure of Puerto Ricans resembled that of Europeans previously reported (Tables 2 and 3).[34]–[36] The continental origin of the IL23R gene in Puerto Ricans was almost entirely of European origin. These results may indicate that NOD2 and IL23R segments of European ancestry contribute to CD in Puerto Ricans. In contrast, we also observed an association with the GALC/GPR65 genes in a region with an excess contribution from the American continent in Puerto Ricans. The significance of this association in Puerto Rican UC was of the same order of magnitude as that of IL23R for CD, but association in European populations is far lower for GALC/GPR65 than for IL23R (www.broadinstitute.org/mpg/ricopili). [3] This difference may be due to Ancient American-derived GALC/GPR65 variation or to European-derived variation in a Puerto Rican “genetic background.”

In addition, we observed some evidence consistent with an association between CD and a SNP in the promoter of the BAZ1A gene, at the end of a region included on the ImmunoChip in order to fine-map a psoriasis susceptibility locus. [37] The observed p-value was not significant when corrected for multiple comparisons but showed some deviation from the null on the qq-plot. The association was located in a region almost completely of American ancestry. Expression studies have demonstrated that BAZ1A is expressed in intestinal tissue (GDS559, GDS2642, GDS3119) as well as altered expression in response to zymosan and to lipopolysaccharide (dbGEO GDS310, GDS2216, GDS2686). The SNP itself alters a binding site for the transcription factors Sp1 (Transfac M000008) and MZF1 (Transfac M00083). This evidence, along with our result, raise the possibility that BAZ1A of American origin contributes to IBD when admixed with European and African continental ancestry. Testing this hypothesis with a more complete fine-mapping study of this gene in populations with American ancestry is therefore warranted.

In conclusion, we have observed that NOD2 and IL23R genomic regions of European descent contribute to CD in Puerto Rico. In addition, our observations suggest that the promise of the study of IBD in non-European populations is realizable: that the study of different genes with different continental ancestries admixed in different proportions will contribute to the understanding of the genetics of IBD in all populations.

## Supporting Information

File S1 **File S1 contains Supplemental Figures, including regional plots of associations and regional plots of local continental ancestry for NOD2, IL23R, GPR65, LCE complex, BAZ1A, and BCAR1/CFDP1, as well as a “qqplot” for BAZ1A.**
(PDF)
