# Supplementary material for: Association of NOD2 and IL23R with Inflammatory Bowel Disease in Puerto Rico
Source: PLoS One. 2014 Sep 26;9(9):e108204. doi: 10.1371/journal.pone.0108204 (PMC4178120; doi:10.1371/journal.pone.0108204)

Supplemental Figures

Association of NOD2 and IL23R with Inflammatory Bowel Disease in Puerto Rico

V Ballester, X Guo, R Vendrell, T Haritunians, AM Klomhaus, D Li,
DPB McGovern, JI Rotter, EA Torres, KD Taylor

Supplementary Figure S1. Association and "local" continental ancestry of previously identified IBD genes: NOD2, IL23R, and GPR65.

Upper graph: association of individual SNPs with IBD in Puerto Rico. Bars at top of graphs show gene regions. Lower graph: proportion of continental ancestry in corresponding region estimated using LAMP-LD trained on African, European, and American haplotypes (determined by fastPHASE; African, dark blue; European, red; American, green).

A, Association of the NOD2 region with CD.

B, Association of the IL23R/IL12RB1 region with CD.

C, Association of the GALC/GPR65 region with UC.

A. Association of the NOD2 region with CD

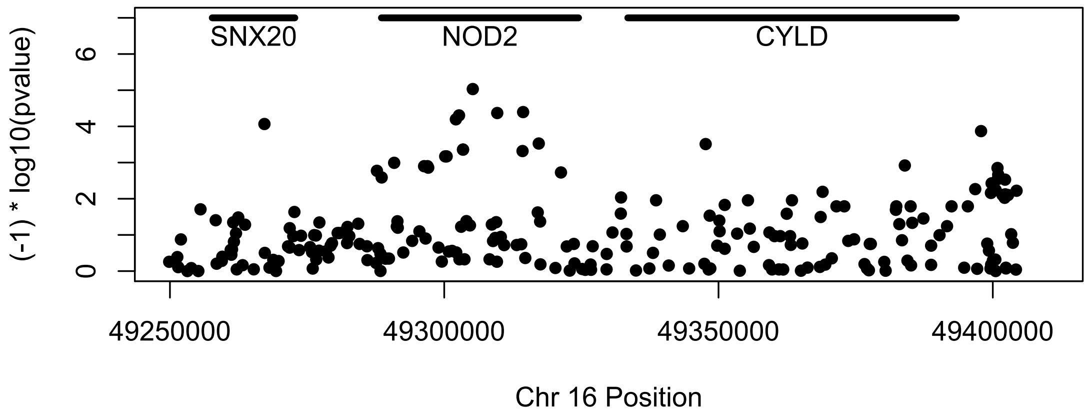

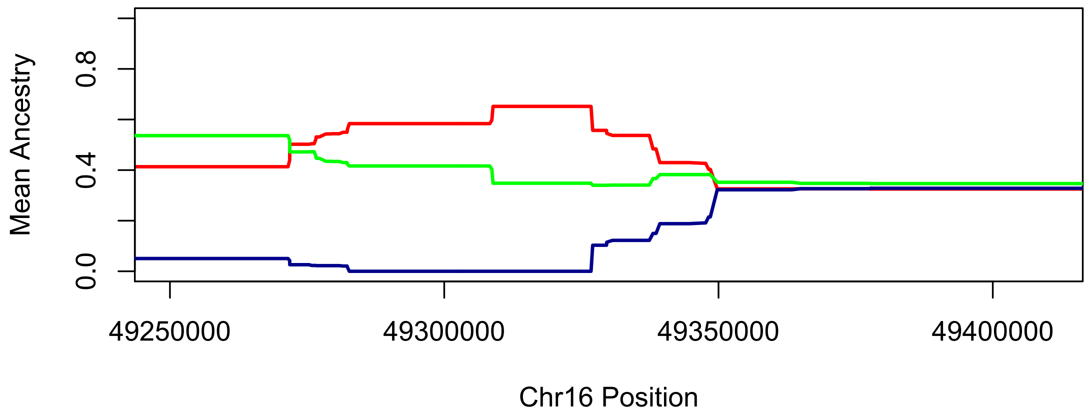

B. Association of the IL23R/IL12RB1 region with CD

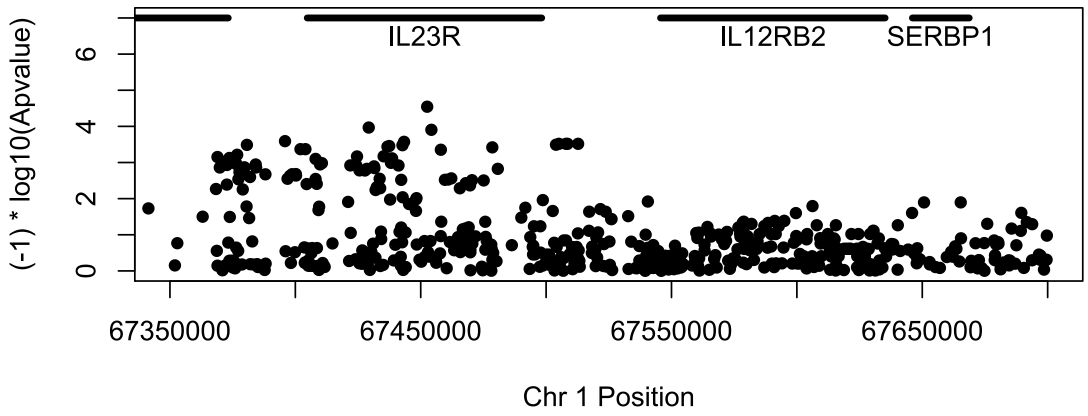

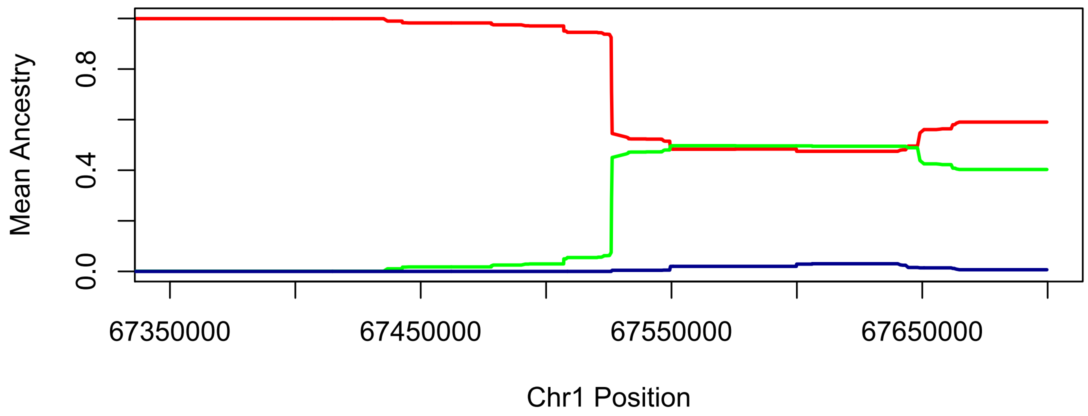

C. Association of the GALC/GPR65 region with UC.

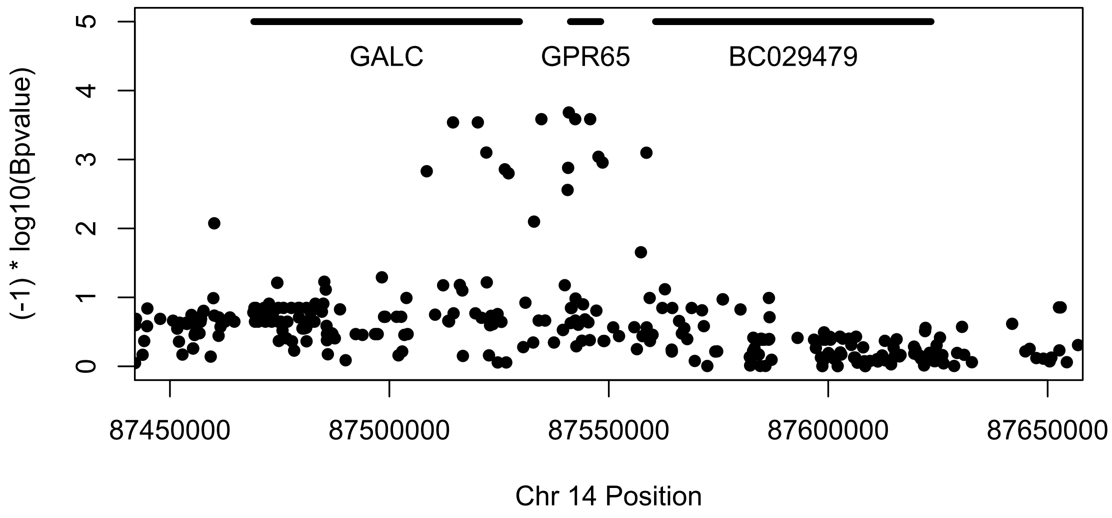

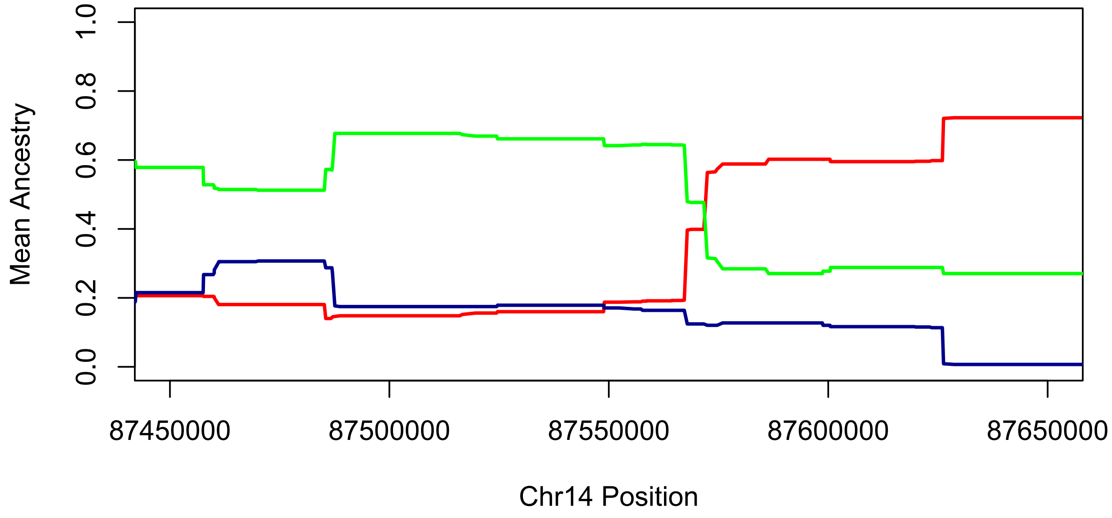

Supplemental Figure S2. Association and “local” continental ancestry of possible IBD genes: LCE1E, BAZ1A, BCAR1/CFDP1.
Upper graph: association of individual SNPs with IBD in Puerto Rico. Bars at top of graphs show gene regions. Lower graph, proportion of continental ancestry in corresponding region estimated using LAMP-LD trained on African, European, and American haplotypes (determined by fastPHASE; African, dark blue; European, red; American, green).
A, Association of the LCE gene complex with IBD.
B, Association of BAZ1A promoter region with CD.
C, Association of the BCAR1/CFDP1 region with UC.

A. Association of the LCE gene complex with IBD.

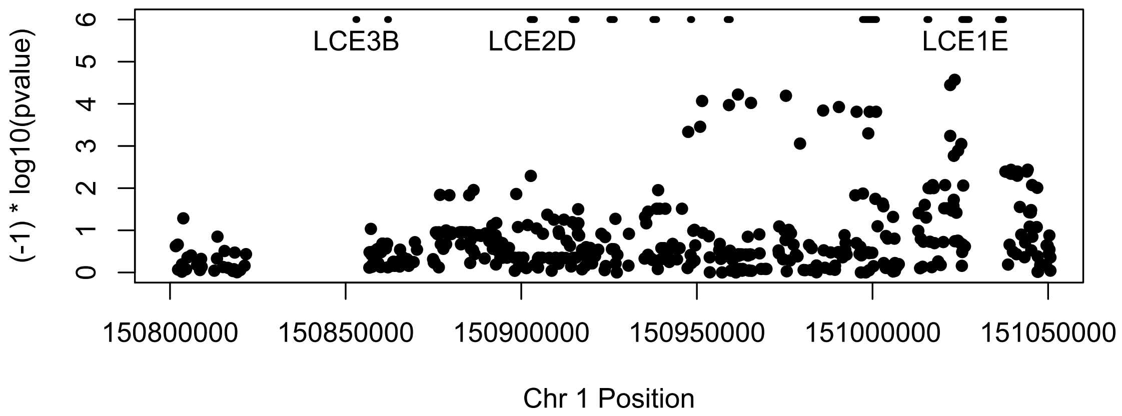

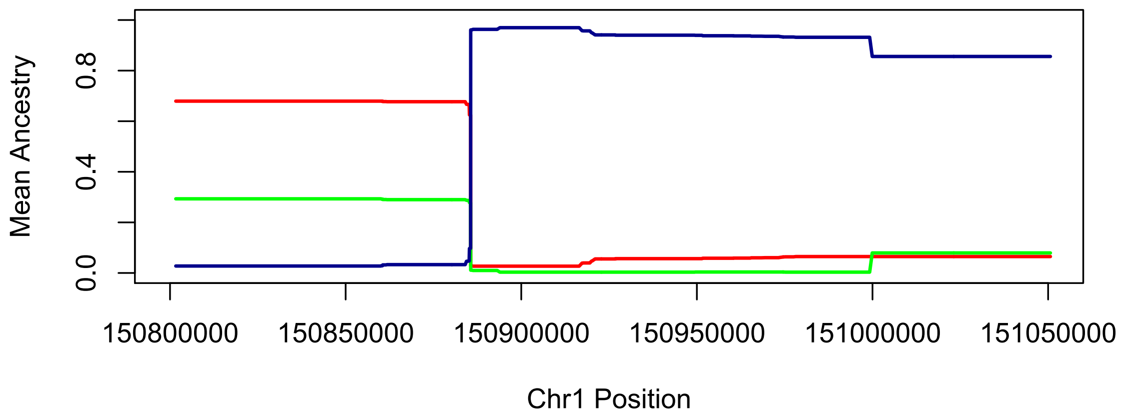

B. Association of BAZ1A promoter region with CD.

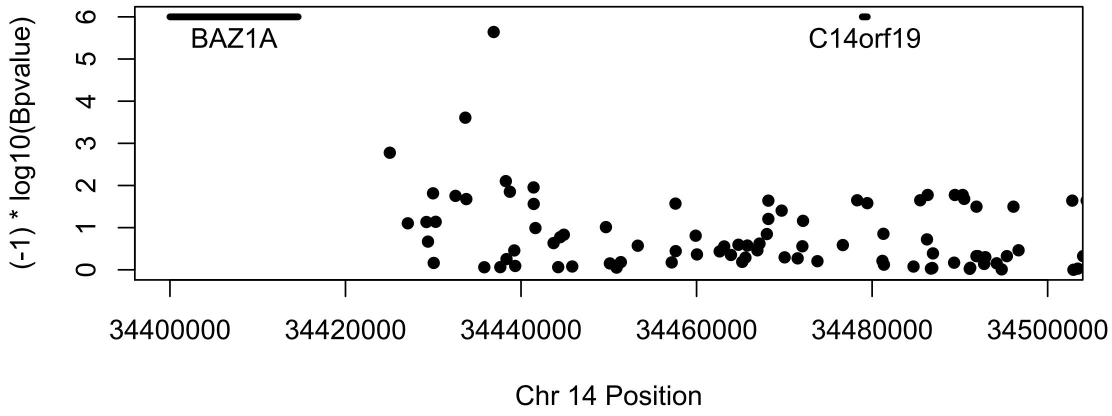

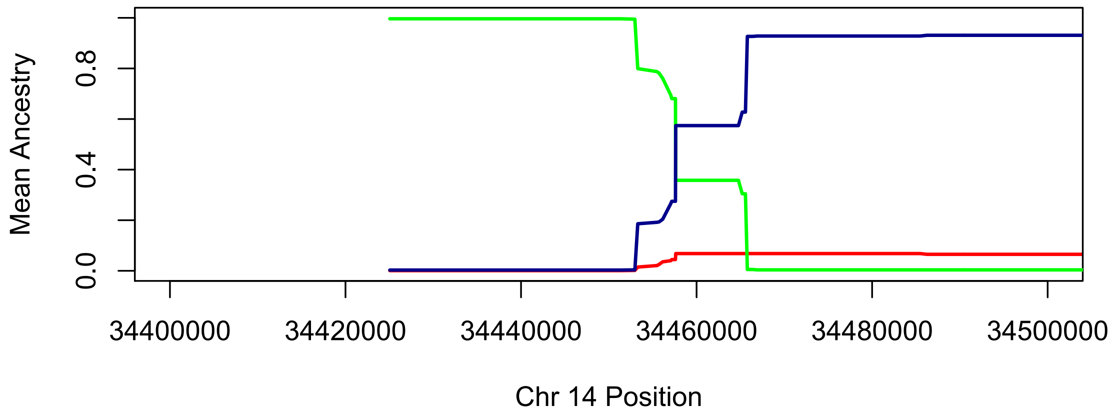

C. Association of the BCAR1/CFDP1 region with UC.

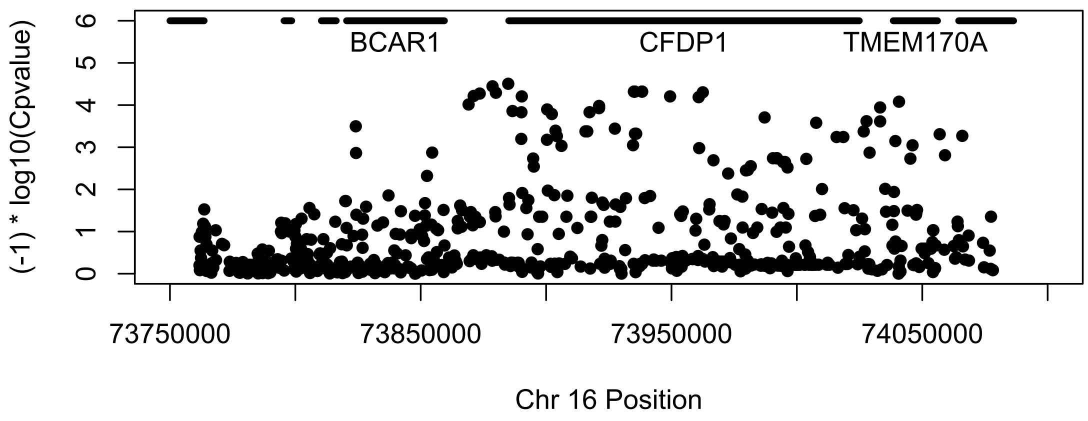

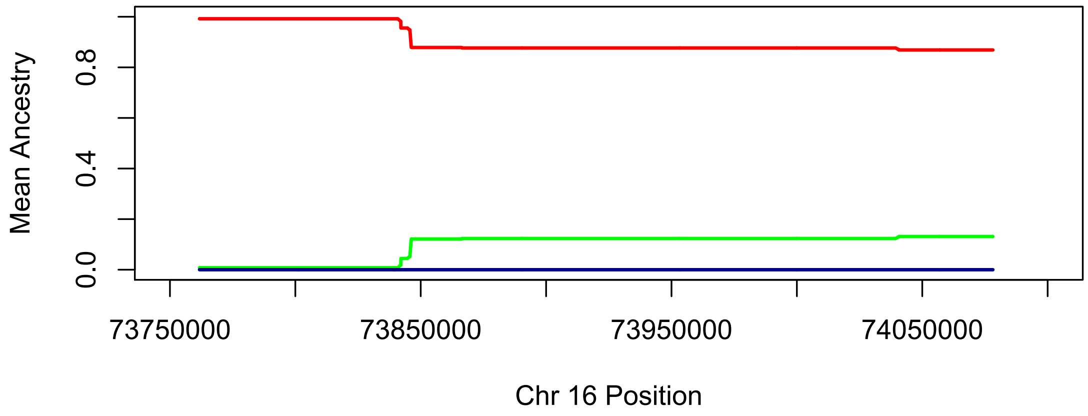

Supplementary Figure 3.

Observed -1 log 10 pvalues plotted versus expected values under the hypothesis of no association. Genomic inflation for this plot was 1.05. A similar plot for UC did not show any deviation of observed from expected significance.

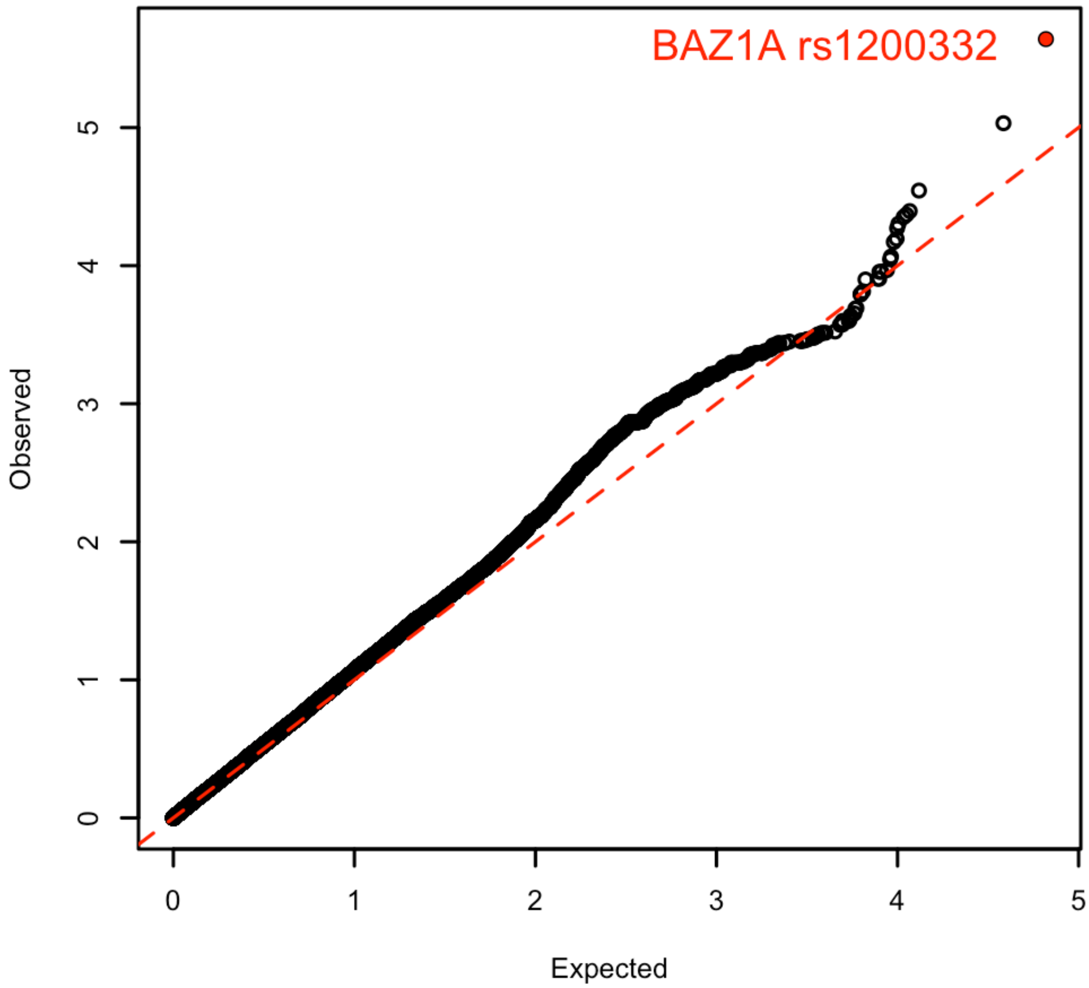

Supplement: File S1 — contains Supplemental Figures, including regional plots of associations and regional plots of local continental ancestry for NOD2, IL23R, GPR65, LCE complex, BAZ1A, and BCAR1/CFDP1, as well as a “qqplot” for BAZ1A. (PDF) [file pone.0108204.s001.pdf]
